# Regional brain metabolism and metabolic connectivity alterations in corticobasal syndrome assessed with [^18^F]FDG-PET

**DOI:** 10.1007/s00259-026-08108-6

**Published:** 2026-08-01

**Authors:** Matilde Nerattini, Andrea Leo, Valentina Bessi, Anna De Rosa, Giovanna De Michele, Valeria Gaudieri, Giulia Giacomucci, Alberto Cuocolo, Giuseppe De Michele, Valentina Berti, Sabina Pappatà

**Affiliations:** 1 Department of Experimental and Clinical Biomedical Sciences, Nuclear Medicine Unit, University of Florence, Florence, Italy; 2 Department of Diagnostic Imaging, Nuclear Medicine Unit, N.O.P. - S. Stefano, U.S.L. Toscana Centro, Prato, Italy; 3 Department of Neuroscience, Psychology, Drug Research and Child Health, University of Florence, Florence, Italy; 4 Department of Neurosciences, Reproductive and Odontostomatological Sciences, Federico II University, Naples, Italy; 5 Department of Advanced Biomedical Sciences, Federico II University, Naples, Italy; 6 IRCCS SYNLAB SDN, Naples, Italy

**Keywords:** Corticobasal syndrome, FDG-PET, Metabolic connectivity, Brain networks, Neurodegeneration

## Abstract

**Purpose:**

Corticobasal syndrome (CBS) is a clinically defined phenotype with different underlying neuropathological substrates most commonly the 4-repeat tauopathy corticobasal degeneration (CBD). 2-[^18^F] fluoro-2-deoxy-D-glucose Positron Emission Tomography ([^18^F]FDG-PET) studies have described regional metabolic abnormalities but detailed subcortical and cerebellar structures involvement is less known and metabolic connectivity remains unexplored. This study combined voxel-based, region-of-interest (ROI) analyses and connectivity approaches to further characterize metabolic alterations and to provide a network-level framework in CBS.

**Methods:**

Thirty-nine CBS patients underwent [^18^F]FDG-PET at two sites, with images flipped to align the most affected hemisphere; 99 controls were drawn from a national normative database. Voxel-based SPM method and ROIs analysis were performed. Thirty-six bilateral cortical and subcortical regions of interest were processed to perform interregional correlation and network-based analyses within five functional networks. Pairwise Spearman correlations were computed from normalized regional signals. Group differences were assessed at regional and network levels using Fisher-transformed correlations, Cohen’s q, permutation testing (10,000 iterations), and graph-theoretical metrics (node strength, clustering coefficient; threshold ρ > 0.25).

**Results:**

CBS patients showed asymmetric hypometabolism predominantly in frontal, parietal, and temporal cortices, caudate and thalamus of the predominantly affected hemisphere, with additional contralateral involvement, notably in the cerebellum and caudate. Metabolic connectivity analyses revealed widespread intra- and inter-network disconnection, particularly involving frontal, parietal, and sensorimotor cortices, and thalamo–cortical pathways, with significant lateralization toward the affected hemisphere. Graph analysis showed decreased cortical node strength with relative increases in subcortical hubs and mixed changes in clustering coefficients, suggesting network reorganization.

**Conclusion:**

This first [^18^F]FDG-PET metabolic connectivity study in CBS demonstrates asymmetric and bilateral regional hypometabolism, widespread and lateralized network disconnection, and subcortical reorganization. These findings reflect both degenerative, functional and compensatory mechanisms and highlight metabolic connectivity as a sensitive marker of network-level alterations in neurodegenerative disease.

## Introduction

Corticobasal syndrome (CBS) is a clinically defined phenotype with different underlying neuropathological substrates. The most frequent neuropathological substrate is corticobasal degeneration (CBD), a rare, progressive 4-repeat (4R) tauopathy characterized by hyperphosphorylated tau accumulation in neurons and glia [1, 2], followed by progressive supranuclear palsy and Alzheimer’s disease [2]. Other neurodegenerative disorders, including Lewy Body disease and TDP-43 Frontotemporal dementia, are less frequently reported [2]. Clinically, CBS is defined by asymmetric motor deficits—rigidity, dystonia, myoclonus, and akinesia—accompanied by higher-order cortical impairments such as apraxia, cortical sensory loss, agnosia, and alien limb phenomena [1]. [^18^F] fluoro-2-deoxy-D-glucose Positron Emission Tomography ([^18^F]FDG-PET) has emerged as a supportive biomarker for CBS diagnosis, in line with European intersocietal recommendations that consider it an essential tool once CBS is clinically suspected [3].

[^18^F]FDG-PET studies have consistently demonstrated predominantly asymmetric patterns of hypometabolism in CBS relative to controls, as reviewed in [4]. Hypometabolism typically involves cortical regions—parietal, prefrontal and posterior frontal cortices, and occasionally temporal and occipital areas—and extends to subcortical structures, most prominently the caudate nucleus and thalamus, contralateral to the most affected body side [4–14]. Bilateral involvement has been also reported [4, 8, 15]. Recent studies, integrating [^18^F]FDG-PET with post-mortem data [7] and with CSF and/or PET amyloid/tau biomarkers [16, 17] have shown that metabolic patterns in CBS vary according to the underlying pathology, with hypometabolism involving the temporoparietal cortex in AD-CBS [7, 8, 16, 17], the medial frontal cortex in PSP-CBS [7, 17], and the frontoparietal cortex, thalamus and caudate in CBD-CBS [7, 17]. Despite these pathology-specific differences, the perirolandic cortex, particularly the precentral gyrus emerges as a shared site of hypometabolism across CBS neuropathological subtypes [7]. However, this finding has not been replicated in AD-CBS where a lack of precentral hypometabolism has been reported [16]. The cerebellum might be involved, although less investigated [18]. Notably, these metabolic changes involve but extend beyond the regions primarily affected by the underlying neuropathology, suggesting that hypometabolism reflects both the neuropathology and functional/degenerative trans-synaptic (diaschisis-like) effects. In tau-related CBS, pathological models propose that tau pathology spreads along functionally connected networks in a prion-like manner, disrupting both neuronal and glial compartments and ultimately affecting distributed brain circuits [19].

Such network-based propagation highlights CBS as a disorder of large-scale brain networks rather than isolated regions, emphasizing the need for analytic approaches that can capture these systems-level alterations. Connectivity analyses are particularly suited for this purpose, as they assess interactions between brain regions and can reveal both degenerative and compensatory processes within affected networks [20].

In CBS, MRI-based connectivity studies—mostly resting-state fMRI—have reported both decreases and increases in functional connectivity in patients compared to controls [21–25]. CBS-specific reductions have been observed between thalamus and several cortical areas, basal ganglia, and cerebellum, whereas increases in connectivity have been documented between the dentate nucleus and cortical regions such as the anterior cingulate cortex, supplementary motor area, primary motor cortex, and prefrontal cortex, predominantly in the most affected hemisphere [22]. Additional widespread changes have been noted in frontoparietal, attention, default mode, salience, and executive-control networks in both directions [21–26].

Unlike MRI, [^18^F]FDG-PET connectivity is based on interregional covariance of glucose metabolism, providing a proxy for synaptic activity and offering some advantages, including higher signal-to-noise ratio and complementary modality-specific information [20]. Despite its relevance for assessing network-level dysfunction, metabolic connectivity has not yet been examined in CBS.

In this study, we use [^18^F]FDG-PET to map regional hypometabolism and investigate metabolic connectivity alterations in CBS, providing a network-level framework to advance understanding of disease pathophysiology.

## Methods

### Subjects

Patients’ characteristics are shown in Table 1. Thirty-nine CBS patients (16 male) were recruited in two different sites: the Neurologic Unit of the Federico II University of Naples and the Neurology Unit of Careggi University Hospital in Florence. Patients had an age at PET (mean +/- SD) of 72.6 +/- 6.1 years. Clinical classes were defined according to Armstrong and colleagues [1] as Probable CBS, if they had asymmetric presentation of two between: (a) limb rigidity or akinesia, (b) limb dystonia (c) limb myoclonus plus two of: (d) orobuccal or limb apraxia, (e) cortical sensory deficit, (f) alien limb phenomena (more than simple levitation), or Possible CBS if they presented (also symmetrically) one of: (a) limb rigidity or akinesia (b) limb dystonia (c) limb myoclonus plus one of (d) orobuccal or limb apraxia, (e) cortical sensory deficit, (f) alien limb phenomena (more than simple levitation) [1]. In total, 19 patients (across the two sites) were defined as Probable CBS and 20 as Possible CBS. Disease duration was 3.4 +/- 1.6 years (mean +/- SD), MMSE (mean +/- SD 22.92 +/- 4.74) and MDS-UPDRS part III (median 35, range 24–54) were taken as additional parameters. This is an observational study. The study was approved by the Ethical Committee of both centers (study code: 29361_DM/2026 and 17/2016). Informed consent was obtained from all individual participants included in the study.

**Table 1:** Patients and controls characteristics

	Patients	Controls
Age, years	72.7 (6.1)	60.6 (13.0)
Gender (M:F)	16:23	44:55
Diagnosis	Possible CBS: 20 Probable CBS: 19	/
Disease Duration, years	3.4 (1.6)	/
MMSE, unitless	22.9/30 (4.7)	29.3 (0.8)
MDS-UPDRS III, unitless	24-54/132 (median 35)	/

Values are means (standard deviations), unless otherwise indicated. Abbreviations: CBS, corticobasal syndrome; M, male; F, female; MMSE, Mini-Mental State Examination; MDS-UPDRS, Movement Disorder Society–Sponsored Revision of the Unified Parkinson’s Disease Rating Scale

The control group consisted of 99 cognitively unimpaired individuals selected from the Italian Association of Nuclear Medicine (AIMN) national normative [¹⁸F]FDG-PET database. Demographic and clinical characteristics are reported in Table 1. The normative database was established through a multicenter collaborative initiative, which also included the two centers participating in the present study. All PET examinations were acquired according to the EANM and national procedural guidelines using standardized acquisition and reconstruction protocols.

Subjects were included in the normative database if they had preserved global cognitive function (MMSE > 28), no evidence of regional hypometabolism on voxel-based statistical analysis, and remained free of neurological disorders during a clinical follow-up of at least one year. The Italian normative database has previously been validated against the major international FDG-PET control databases [27].

### PET image acquisition and preprocessing

Patients from both sites underwent brain [^18^F]FDG-PET with a standardized acquisition protocol in accordance with the national and European guidelines [3]. Patients were injected with 185–200 MBq of [^18^F]FDG and remained in a dimly lit, quiet room for 30–40 min after injection, with instructions to keep their eyes closed. PET acquisition began between 30 and 45 min post-injection and lasted for 15 min. Brain images were acquired in 3D mode using a Philips Time-of-flight (TF) PET/CT scanner (Gemini TF 16 and Ingenuity) and a GE PET/CT (Discovery LS) system, as previously described [28, 29]. Images were reconstructed using 3D iterative algorithm and corrected for attenuation using the maps derived from CT data. They were then manually reoriented along the commissural plane, normalized to the Montreal Neurological Institute (MNI) space using dementia-specific [^18^F]FDG-PET template [30], resampled to 2 mm isotropic voxels, and smoothed with an 8 mm FWHM Gaussian kernel in statistical parametric mapping (SPM12) [31]. Control images from the normative database underwent the same preprocessing pipeline, including spatial normalization using the dementia-specific [¹⁸F]FDG-PET template. Given the asymmetrical presentation of the disease, scans with prominent hypometabolism in left hemisphere (corresponding to patients with motor signs in right body side) were flipped, so that the most affected hemisphere would be the right one.

### SPM analyses

All [^18^F]FDG-PET data were analyzed using statistical parametric mapping (SPM12) [28] on MATLAB (MathWorks Inc, Sherborn, MA, USA). Comparisons between groups were performed using independent samples T-test design, including the group of controls, using age and center as nuisance variables and proportional scaling to white matter activity. Quality control included exclusion of subjects with relevant white matter abnormalities on MRI or CT and visual inspection of the white matter masks after spatial normalization to ensure correct anatomical positioning. The significance threshold was set at *p* < 0.05, Family-Wise Error (FWE)-corrected for multiple comparisons at the voxel level. Only clusters containing more than 20 voxels were deemed to be significant.

### ROIs definition and connectivity estimation

First, a set of ROIs was defined by choosing relevant regions that covered cortex and subcortical structures. We selected 36 bilateral ROIs and, for each of them, created a 3D mask using WFU PickAtlas [32], plus additional masks for white matter and for total brain volume. Region masks were resampled to match the resolution of normalized PET images and used to extract the signal for each participant, as the average across all the voxels in each ROI. Signal extraction was performed with the MarsBaR toolbox [https://marsbartoolbox.github.io/download.html*]*, running on SPM12.

The average voxel counts calculated for each ROIs were scaled as a percentage of the white matter signal and processed for group comparison and interregional correlation analysis. For this latter, two subjects x ROIs matrices were generated, whose size was therefore 99 × 36 for controls and 39 × 36 for CBS patients for the whole brain analysis. All matrices underwent a z-score normalization within columns, so making data from the same region have mean equal to zero and standard deviation equal to 1. Connectivity matrices were then estimated by computing all the pairwise correlations between regions (Spearman’s ρ), thus obtaining two 36 × 36 matrices for the whole-brain analysis, and, for the network-based analysis, five squared matrices whose size was equal to the number of ROIs comprised in each network. These matrices were used in the subsequent group analyses.

### Group analysis

Group comparison was first performed to assess regional metabolic differences between CBS patients and controls, using a General Linear Model (GLM). For each ROI, metabolic values scaled to WM were entered as the dependent variable, disease group (CBS vs. controls) as the fixed factor of interest, and acquisition center as a categorical covariate. The effect of disease group was therefore estimated after adjusting for potential center-related variability. Statistical significance was assessed using the F statistic associated with the disease group term. To account for multiple comparisons across ROIs, p-values were corrected using the Benjamini–Hochberg false discovery rate (FDR), with statistical significance set at FDR-corrected *p* < 0.05.

To measure how connectivity could differ as a result of CBS, we examined metabolic connectivity differences using the same set of ROIs. For each group whole-brain regional correlation matrices were converted to distances (i.e., 1 - Spearman’s ρ), and similarity was defined as the correlation (Spearman’s ρ) between the two distance matrices. This result was nonparametrically tested for significance against the null distribution from a bootstrap test (10,000 iterations).

Then, we looked for network-level group differences: covariance matrices were converted to z-scores using the Fisher transformation, and the values in each of the five matrices, each belonging to a different network, were compared between groups using two-sample t-tests. In addition, we computed interhemispheric connectivity by considering separately the ROIs from left and right hemispheres and performing a two-way ANOVA with Group (controls and CBS) and Side (Left and Right) as factors.

To further assess connectivity differences at a regional level, we relied on Cohen’s q, a measure of effect size that can be defined as the difference between two z-transformed correlation values. Matrices of q values were therefore obtained by subtracting the Fisher-transformed matrices from controls and patients, and indicated the regions whose connectivity was affected by CBS. This analysis was performed for whole-brain and network-level matrices.

Statistical significance was assessed with a permutation test: the subject x ROIs matrices were shuffled row-wise 10.000 times (i.e. shuffling the order of subjects in each ROI) and a set of null connectivity matrices was then computed by computing the pairwise correlations (Spearman’s ρ, as described above). Afterwards, we applied the Fisher transformation also to the null matrices and computed a distribution of 10.000 null Cohen’s q values. Real q values were compared to null distribution, and converted to p-values with a rank test. All statistical analyses were performed with MATLAB R2022b (The MathWorks Inc., 2022).

Statistically significant thresholds were set at *p* ≤ 0.01 for the ROIs comparison and at *p* < 0.01 for connectivity analysis.

### Brain connectivity graph analysis

In addition to the analyses reported above, we computed two graph theory measures, i.e. Node Strength (NS) and Clustering Coefficient (CC) on the connectivity matrices from CBS patients and controls. Node strength can be defined as the sum of weights of links connected to a region (node), being therefore an expression of the extent to which a region is connected to the remainder of the network. Clustering coefficient (CC) is the degree to which regions (nodes) tend to cluster together, and increases with the fraction of node’s neighbors that are neighbors of each other [33]. Connectivity network measures were estimated on the 36 × 36 matrices, after the application of an arbitrary threshold that, according to previous reports on the same topic, was set to 0.25. Analysis was performed with the Brain Connectivity Toolbox [34], running on MATLAB R2022b.

## Results

### SPM analyses

**Fig. 1:**
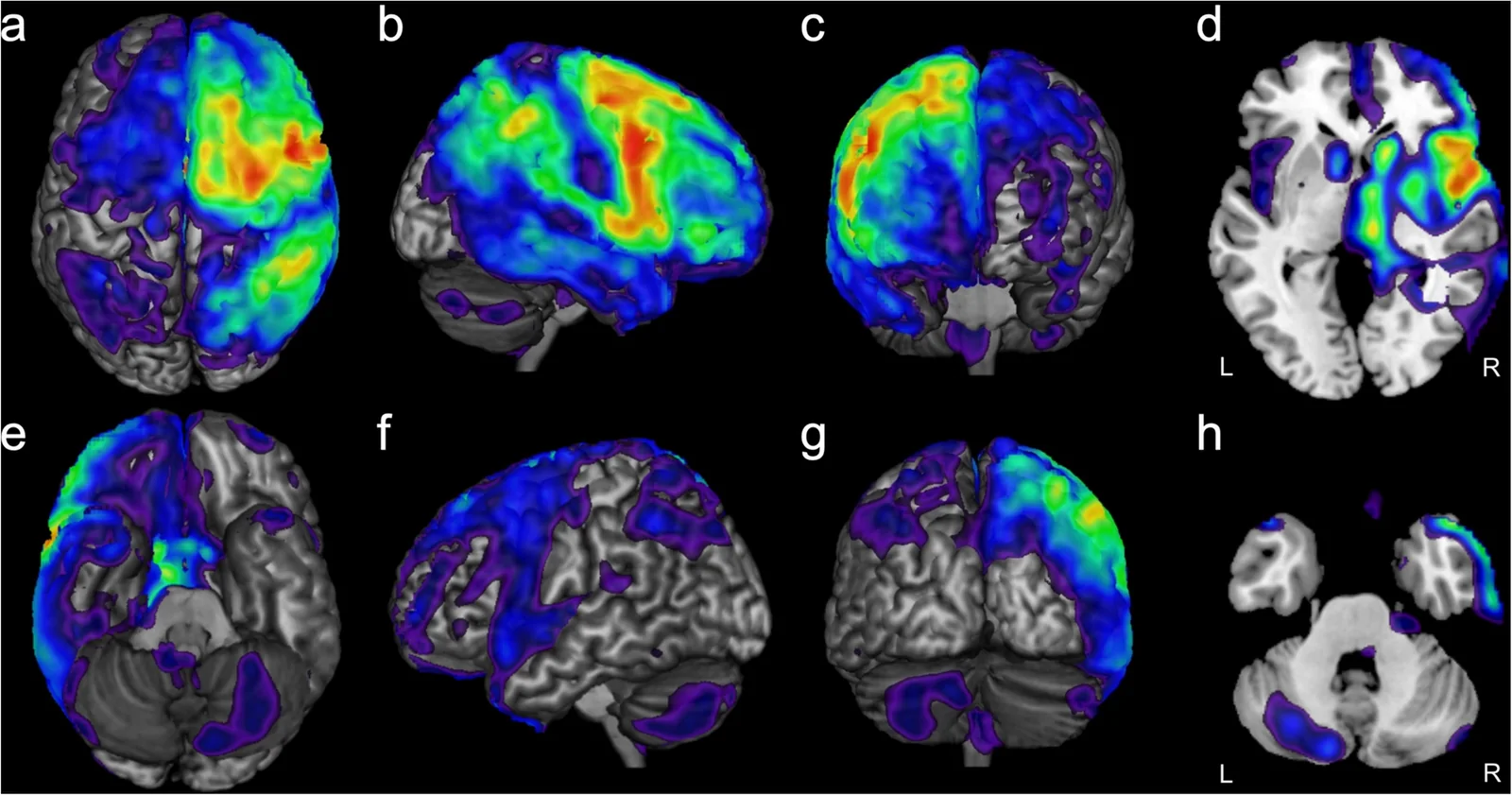
SPM T maps showing significant hypometabolism in CBS patients as compared to controls. Maps are represented on color-coded scale and displayed onto a standardized surface rendered MRI (a, b, c, e, f, g) and onto a standardized axial MRI (d, h, neurological orientation)

Results are found in Table 2; Fig. 1. Controlling for age, CBS patients showed significant hypometabolism compared to controls mostly, but not exclusively, in the right (i.e. most affected) hemisphere. Specifically, patients exhibited highly significant and more marked hypometabolism in the right precentral and paracentral lobules, superior parietal lobule, precuneus, anterior and posterior cingulate gyrus, middle and inferior frontal gyri, caudate body; and, to a lesser extent, in the inferior temporal gyrus as well as in the left superior parietal lobule, insula, precentral gyrus, postcentral gyrus, superior frontal gyrus, and cerebellum (*P*_*FWE*_ ≤ 0.034).

**Table 2:** Voxel-based significant reductions in glucose metabolism in CBS vs. controls

Cluster extent (voxels)	*P*_FWE_*	T	Z	*P* uncorrected	MNI coordinates x, y, z (mm)	Anatomic region (AAL)
63,094	< 0.001	12.15	Inf	< 0.001	18 − 12 16	Right Insula
	< 0.001	11.44	Inf	< 0.001	6 − 2 32	Right Cingulate Gyrus
	< 0.001	11.24	Inf	< 0.001	14 − 8 32	Right Paracentral Lobule
	< 0.001	11.00	Inf	< 0.001	18 6 14	Right Caudate Body
	< 0.001	10.51	Inf	< 0.001	50 8 36	Right Middle Frontal Gyrus
	< 0.001	10.36	Inf	< 0.001	16–22 32	Right Posterior Cingulate
	< 0.001	10.10	Inf	< 0.001	30 − 6 54	Right Superior Parietal Lobule
	< 0.001	9.79	Inf	< 0.001	62 10 22	Right Inferior Frontal Gyrus
	< 0.001	9.55	Inf	< 0.001	58 14 6	Right Precentral Gyrus
	< 0.001	9.32	Inf	< 0.001	22 30 56	Right Superior Parietal Lobule
	< 0.001	9.28	Inf	< 0.001	24 2 62	Right Precuneus
	< 0.001	9.23	Inf	< 0.001	50 0 0	Right Insula
106	0.001	5.68	5.36	< 0.001	36 8–50	Right Inferior Temporal Gyrus
159	0.002	5.55	5.25	< 0.001	−40 −48 44	Left Superior Parietal Lobule
362	0.002	5.50	5.21	< 0.001	−40 20 − 16	Left Insula
	0.002	5.47	5.18	< 0.001	−46 −4 −2	Left Precentral Gyrus
84	0.002	5.47	5.18	< 0.001	−18 −24 82	Left Postcentral Gyrus
30	0.007	5.19	4.94	< 0.001	−34 64 2	Left Superior Frontal Gyrus
178	0.034	4.60	4.42	< 0.001	2–40 −44	Cerebellum Vermis
522	0.001	4.47	4.31	< 0.001	−12 −82 −34	Left Cerebellum Crus
		4.32	4.17	< 0.001	−30 −48 −50	Left Cerebellum
		4.29	4.14	< 0.001	−32 −74 −40	Left Cerebellum

Table shows cluster extent, statistics, coordinates and location of main peaks of significant clusters. ^*^P < *0*.*05* corrected for Family-Type Wise Error (FWE). Analyses were adjusted by age and global mean FDG uptake. Abbreviations: AAL, Automated Anatomical Labeling; MNI, Montreal Neurological Institute

### ROI analyses

Results are found in Table 3. ROI analysis demonstrated significant reductions of cerebral metabolic activity in most brain regions of the right hemisphere and in several regions of the left hemisphere in CBS patients compared to controls. In the right (most affected) hemisphere, CBS patients showed significantly reduced metabolic activity in the prefrontal cortex, Brodmann areas (BA) 6, 7, and 8, anterior cingulate cortex, primary motor and sensory cortices, lateral parietal cortices, posterior cingulate cortex, and lateral temporal cortices, as well as in several subcortical regions, including the hippocampus, caudate nucleus, putamen, and thalamus. In the left hemisphere, CBS patients showed a less widespread but still significant reduction in metabolic activity, involving the prefrontal cortex, BAs 6, 7, and 8, anterior cingulate cortex, lateral parietal cortices, posterior cingulate cortex, caudate, putamen, and thalamus.

**Table 3:** ROIs-based significant reductions in glucose metabolism in CBS vs. controls

	CBS	Controls	Sig. FDR-corr
PreFrontal L	1.83 ± 0.25	1.97 ± 0.19	*p* = 0.00392
PreFrontal R	1.82 ± 0.23	2.15 ± 0.22	*p = 9.56038 E-09*
BA6 L	2.08 ± 0.37	2.24 ± 0.22	*p* = 0.0125
BA6 R	1.91 ± 0.38	2.31 ± 0.24	*p = 1.06912 E-08*
BA8 L	1.78 ± 0.27	2.01 ± 0.20	*p = 1.85964 E-05*
BA8 R	1.77 ± 0.33	2.21 ± 0.25	*p = 7.35027E-10*
ACC L	1.89 ± 0.23	2.11 ± 0.20	*p = 3.90657 E-05*
ACC R	1.83 ± 0.26	2.19 ± 0.23	*p = 9.22161 E-09*
Primary Motor L	2.40 ± 0.46	2.41 ± 0.25	*p* = 0.807
Primary Motor R	2.18 ± 0.42	2.37 ± 0.24	*p* = 0.00457
Primary Sensory L	2.28 ± 0.40	2.33 ± 0.25	*p* = 0.534
Primary Sensory R	2.09 ± 0.38	2.34 ± 0.24	*p = 7.0593 E-05*
BA7 L	2.36 ± 0.44	2.49 ± 0.26	*p* = 0.0728
BA7 R	2.19 ± 0.48	2.50 ± 0.27	*p* = 0.000159
Parietal Lateral L	2.13 ± 0.35	2.27 ± 0.23	*p* = 0.0233
Parietal Lateral R	1.95 ± 0.34	2.36 ± 0.25	*p = 2.46514E-09*
PCC L	2.33 ± 0.31	2.54 ± 0.26	*p* = 0.00782
PCC R	2.30 ± 0.33	2.65 ± 0.29	*p = 1.96619 E-05*
Lateral Temporal L	2.09 ± 0.30	2.13 ± 0.19	*p* = 0.576
Lateral Temporal R	2.00 ± 0.29	2.24 ± 0.21	*p = 2.79666 E-05*
Hippocampus L	1.66 ± 0.18	1.67 ± 0.12	*p* = 0.970
Hippocampus R	1.57 ± 0.18	1.69 ± 0.12	*p* = 0.000671
Amygdala L	1.72 ± 0.23	1.68 ± 0.11	*p* = 0.288
Amygdala R	1.59 ± 0.20	1.64 ± 0.13	*p* = 0.419
Occipital L	2.31 ± 0.39	2.30 ± 0.25	*p* = 0.723
Occipital R	2.23 ± 0.40	2.34 ± 0.26	*p* = 0.288
Caudate Nucleus L	2.31 ± 0.25	2.80 ± 0.32	*p = 3.732 7E-09*
Caudate Nucleus R	1.97 ± 0.31	2.75 ± 0.33	*p = 1.82897 E-16*
Putamen L	2.60 ± 0.30	2.78 ± 0.29	*p* = 0.0156
Putamen R	2.57 ± 0.28	2.96 ± 0.30	*p = 1.74684 E-07*
Thalamus L	2.22 ± 0.22	2.36 ± 0.21	*p* = 0.0155
Thalamus R	1.82 ± 0.16	2.30 ± 0.23	*p = 7.50164 E-16*
Dentate Nucleus L	1.88 ± 0.16	1.88 ± 0.14	*p* = 0.332
Dentate Nucleus R	1.88 ± 0.16	1.88 ± 0.14	*p* = 0.576
Cerebellar Cortex L	2.04 ± 0.29	2.09 ± 0.17	*p* = 0.799
Cerebellar Cortex R	2.12 ± 0.31	2.12 ± 0.17	*p* = 0.807

ACC, anterior cingulate cortex; BA, Brodmann area; L, left; PCC, posterior cingulate

Cortex; R, right

### Metabolic connectivity analyses

#### Regional analyses

**Fig. 2:**
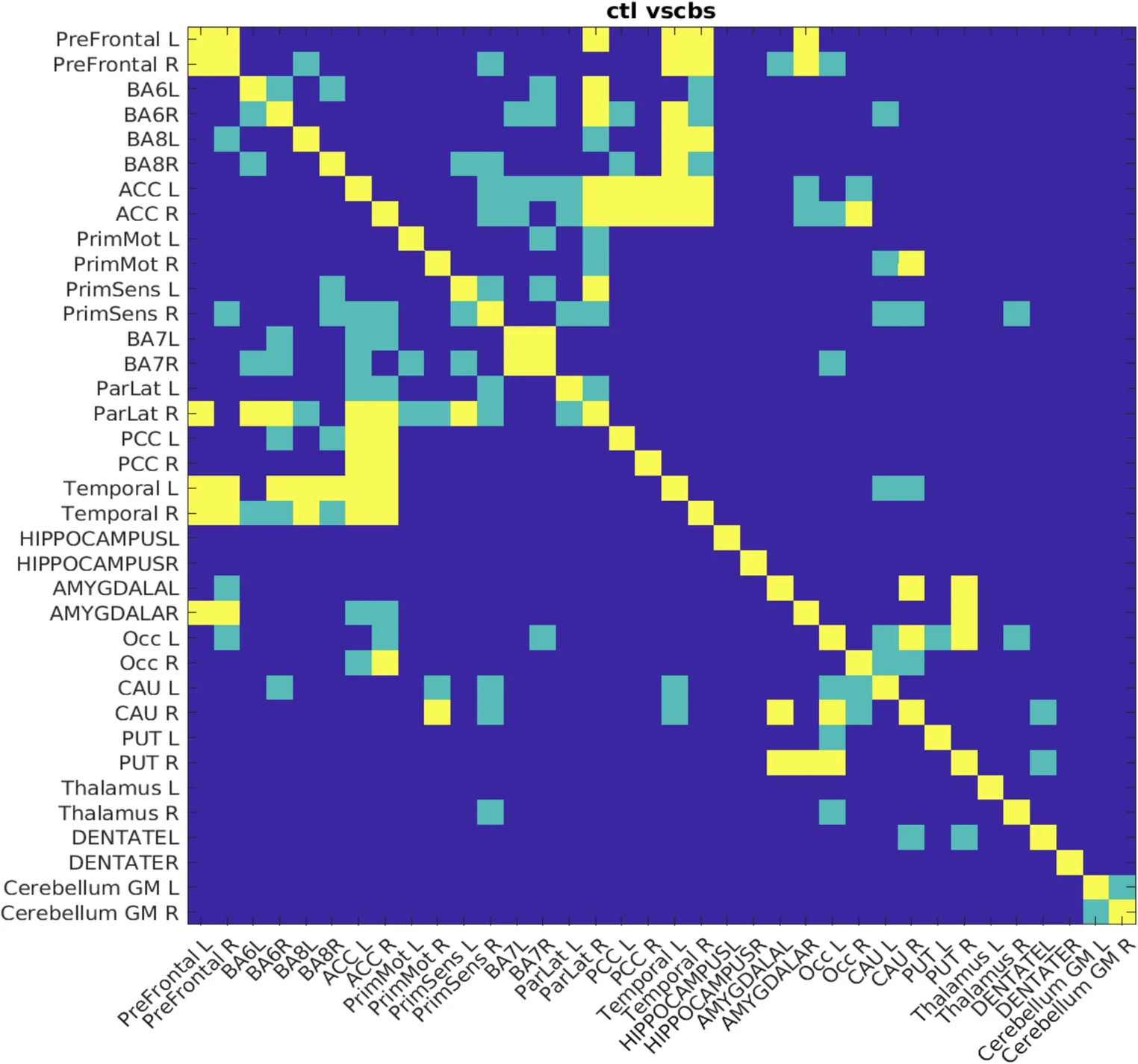
Regional metabolic connectivity reductions in CBS patients compared to controls. Regional correlation matrix illustrating significant reductions in metabolic connectivity in CBS patients relative to controls across cortical and subcortical regions. A dual-threshold approach was applied, with light blue boxes denoting *p* < 0.01 (two-tailed) and yellow boxes denoting *p* < 0.05. Matrix diagonal is in yellow

Regional analysis (Fig. 2) showed widespread reductions in interregional correlations, most prominently involving regions of the most affected hemisphere. We here report in Result section only correlations surviving the conservative threshold of *p* < 0.01 for this hemisphere, while in Fig. 2 we also report results at the less conservative threshold (*p* < 0.05) for both hemispheres.

##### Frontal cortex (prefrontal, ACC)

Prefrontal cortex exhibited diminished coupling with ipsilateral sensorimotor and contralateral BA8, temporal and occipital regions, while the ACC had reduced connectivity with the ipsilateral somatosensory and contralateral parietal, occipital and limbic (amygdala) regions.

##### Parietal cortex (lateral parietal, BA7)

The lateral parietal cortex showed reduced connectivity with the ipsilateral primary sensory, bilateral primary motor cortices, and contralateral parietal and BA8 regions whereas the precuneus exhibited reduced connectivity with bilateral BA6 as well as the contralateral ACC, primary motor and sensory cortices, and occipital cortex.

##### Temporal cortex (lateral temporal, amygdala)

The lateral temporal cortex showed reduced connectivity with bilateral BA6 and contralateral BA8, whereas the amygdala displayed decreased connectivity with bilateral anterior cingulate.

*Occipital cortex* exhibited decreased connectivity with contralateral ACC and bilateral caudates.

##### Sensory-motor cortical network (BA8, BA6, primary motor and sensory cortices)

Within this network, sensorimotor region exhibited the greatest number of significantly reduced correlations (*n* = 10 ROIs) including decreased connectivity with the contralateral homologous area, the ipsilateral frontal, bilateral parietal, the anterior cingulate, the caudate nucleus and the ipsilateral thalamus. BA 8 and BA6 exhibited connectivity reductions with their contralateral homologous areas, the ipsilateral temporal cortex and the contralateral PCC. Additionally, B8 showed reduced connectivity with the bilateral sensorimotor cortex while BA6 demonstrated reduced connectivity with bilateral precuneus. The primary motor cortex displayed reduced correlations with the ipsilateral parietal cortex and the contralateral caudate.

##### Subcortical regions and cerebellum

The caudate and thalamus showed reduced connectivity with cortical regions, specifically the caudate with the ipsilateral primary sensory cortex and the thalamus with the contralateral occipital cortex. Both the caudate and putamen showed decreased connecitivty with the contralateral cerebellar dentate nucleus. The cerebellar cortex exhibited a loss of interhemispheric connectivity.

##### Interhemispheric connectivity

Interhemispheric connectivity resulted significantly different, between patients and controls with a Group effect (i.e., CBS< controls, F = 194.55, Tukey t = 13.67, *p* < 0.0001), a Side effect (Right< Left, F = 25.26, Tukey T = 4.925, *p* < 0.0001) and a significant Side x Group interaction (F = 32.45, *p* < 0.0001).

#### Graph analysis

##### Node Strength

**Fig. 3:**
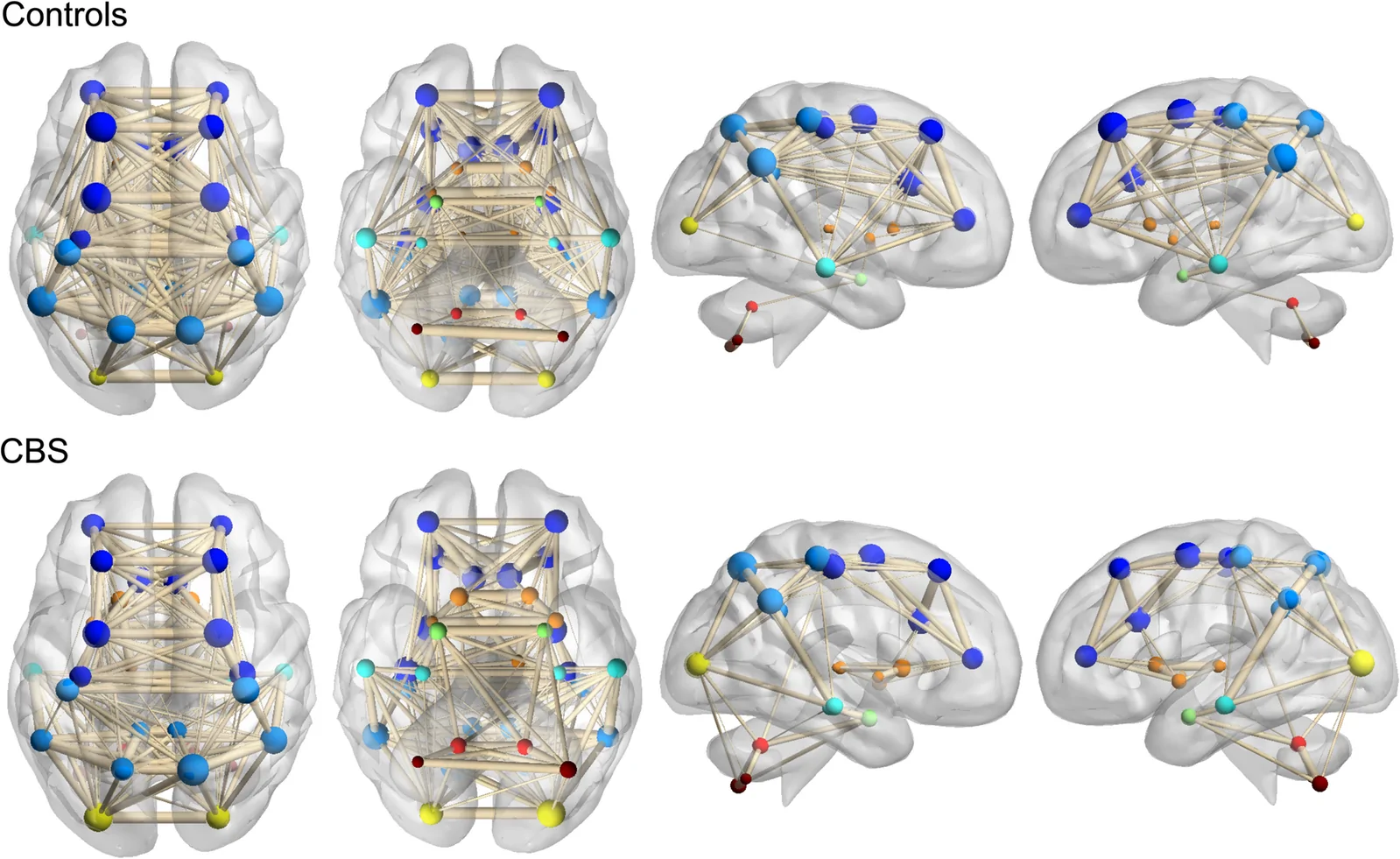
Node strength in healthy controls and CBS patients. Graph theory results showing the distribution of node strength in controls (upper row) and patients with CBS (lower row). Node strength was computed from the connectivity matrices after applying a threshold of 0.25. Node size reflects the magnitude of the metric, while edge thickness indicates the connection strength between two regions in the original connectivity matrix (Spearman’s ρ), after excluding values below 0.25. Nodes color represents brain region: frontal lobe (dark blue), parietal lobe (blue), lateral temporal node (light blue), medial temporal nodes (green), occipital node (yellow), subcortical nuclei (orange), dentate nuclei (red), cerebellar cortex (dark red)

Results are found in Fig. 3. Compared to controls, CBS patients exhibited a strength reduction in cortical nodes mainly of the right hemisphere, and in particular in frontal (prefrontal cortex, including BA6), and parietal (BA7, lateral parietal and primary sensory), as well as in occipital lobe and ACC. Subcortical regions demonstrate a relative increase in node strength, particularly in hippocampus, amygdala, bilateral caudate, bilateral dentate, right putamen, right thalamus and right cerebellum.

##### Clustering Coefficient

**Fig. 4:**
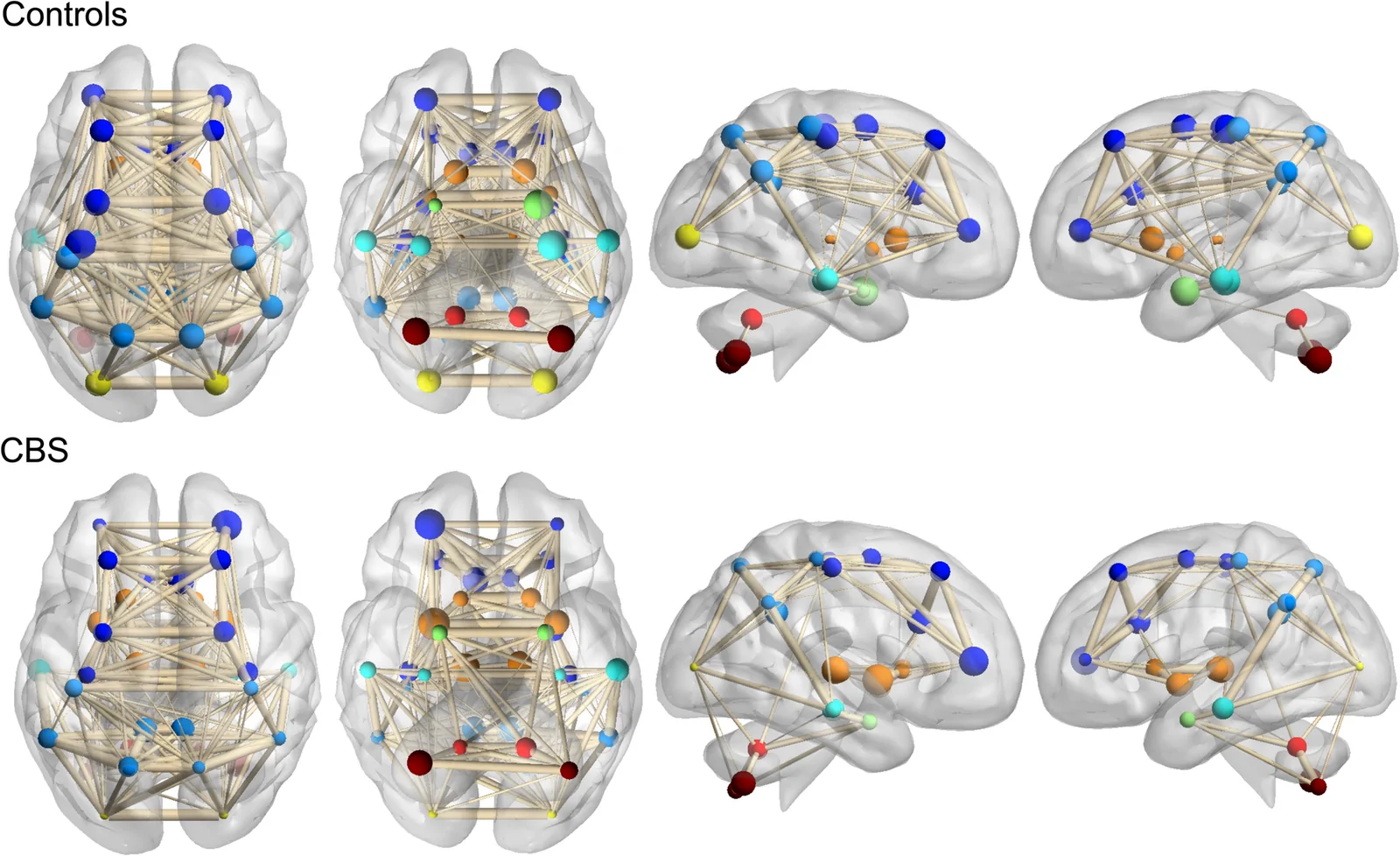
Clustering Coefficient in healthy controls and CBS patients. Graph theory results showing the distribution for clustering coefficients in controls (upper row) and patients with CBS (lower row). Clustering coefficient was computed from the connectivity matrices after applying a threshold of 0.25. Node size reflects the magnitude of the metric, while edge thickness indicates the connection strength between two regions in the original connectivity matrix (Spearman’s ρ), after excluding values below 0.25. Node size reflects the magnitude of the metric, while edge thickness indicates the connection strength between two regions in the original connectivity matrix (Spearman’s ρ), after excluding values below 0.25. Nodes color represents brain region: frontal lobe (dark blue), parietal lobe (blue), lateral temporal node (light blue), medial temporal nodes (green), occipital node (yellow), subcortical nuclei (orange), dentate nuclei (red), cerebellar cortex (dark red)

Results are found in Fig. 4. In CBS, as compared to controls, there were mixed changes in clustering coefficients, toward decreases in the right hemisphere in primary motor cortex, hippocampus, amygdala, occipital, and cerebellum; while toward increases in thalamus and putamen, bilaterally.

## Discussion

This study provides a comprehensive characterization of brain hypometabolic patterns and connectivity alterations in CBS using voxel-wise, ROI-based, and metabolic connectivity analyses of [^18^F]FDG-PET data. We identified asymmetric cortical and subcortical hypometabolism predominantly involving the most affected hemisphere, with additional involvement of some regions in the less affected hemisphere, including subcortical regions. Metabolic connectivity analyses revealed widespread within- and between-cortical network connectivity reductions, most notably in frontal, parietal, and sensorimotor regions, together with decreased cortico-subcortical coupling and cerebellar disconnection. Graph-theoretical measures indicated reduced cortical node strength and increased subcortical hub centrality, suggesting subcortical network reorganization.

We observed an asymmetric pattern of hypometabolism predominantly affecting the right-hemisphere, involving the frontal, parietal, temporal, and cingulate cortices, as well as subcortical structures, particularly the caudate, thalamus. There was also a less extensive but significant contralateral hypometabolism including involvement of cerebellar gray matter. These findings confirm and extend previous literature showing that the most consistent [^18^F]FDG-PET metabolic pattern in CBS is asymmetrical hypometabolism, contralateral to clinical symptoms, encompassing the basal ganglia, thalamus, and cortical regions, including sensorimotor and frontoparietal associative cortices [4–7, 9–13]. In keeping with prior reports [4–6, 8–10, 12, 14], we also observed hypometabolism in additional regions, including temporal, occipital, insular, and cingulate cortices. Notably, at voxel-based analyses we observed cortical cerebellar hypometabolism contralateral to the most affected hemisphere, a less frequently described finding that may reflect trans-synaptic effects consistent with mechanisms of diaschisis. Although originally reported in stroke, crossed cerebellar diaschisis (CCD) is increasingly recognized in neurodegenerative conditions and in CBS [18].

Overall, our results align with the view that CBS hypometabolic patterns partly reflect primary tau deposition, reported post-mortem in CBD [35, 36] and in vivo in CBS using PET-tau studies [37], mainly affecting the basal ganglia, the pre- and post-central gyri, and the superior frontal and parietal gyri. However, findings indicating that hypometabolism is more widespread than tau deposition and extends beyond regions of primary tau pathology [38] suggest that functional disconnection or secondary degeneration within interconnected networks likely contributes to these metabolic changes. Furthermore, considering the diverse neuropathological substrates of CBS, distinct regional metabolic signatures might emerge in relation to the specific molecular profiles, including both amyloid and tau burden [7, 8, 16, 17]. This supports the conceptualization of corticobasal syndrome (CBS) as a network-level disorder [4].

Among the subcortical regions, our analyses revealed a bilateral involvement of caudate nuclei. This is consistent with prior reports of bilateral caudate hypometabolism [4, 8, 15] and with a multimodal study describing lateralized cortical hypometabolism but minimal asymmetry in DAT availability in CBS patients [6], the latter suggesting subtle early bilateral basal ganglia pathological involvement. A more atypical pattern of symmetric hypometabolism affecting bilateral sensorimotor cortices has also been reported [5].

To our knowledge, this is the first study to assess metabolic connectivity in CBS. Previous investigations have primarily employed resting-state fMRI [26], reporting widespread network disruptions characterized by both decreased and increased connectivity [21–25]. Our metabolic connectivity analyses revealed extensive reductions in both intra- and inter-regional correlations within cortical networks, particularly involving frontal, parietal, and sensorimotor cortices, with significant lateralization to the most affected hemisphere, consistent with previous MRI-based connectivity studies [21, 39]. The fronto-parietal-sensorimotor network disconnection likely underlies impaired sensorimotor integration and motor control of the limbs contralateral to the most affected hemisphere in CBS. Evidence from task-based fMRI studies showing reduced activation of motor and parietal regions contralateral to the affected limb further supports the functional relevance of these disruptions [23, 40]. Connectivity reductions were not limited to the fronto-parietal–sensorimotor network, classically implicated in CBS, but also extended to prefrontal, temporal, occipital, limbic regions and precuneus, which might be implicated in sensorimotor processing and cognitive integration in CBS [21–23].

Overall, our results pointed to widespread cortical connectivity reductions in CBS patients. The disruptions of fronto-parietal-sensorimotor network may be consistent with proposed models of tau propagation in 4R tauopathies, including CBD, where tau accumulation leads to neuronal dysfunction. If primary pathology arises in subcortical regions such as the caudate or dentate nucleus, tau has been proposed to propagate in a prion-like manner across functionally connected neurons. Tau-PET studies have shown extensive cortical involvement along functional connectivity pathways, suggesting that the prominent frontal and parietal disconnection observed here may mirror both the topography and propagation routes of tau pathology [24, 25]. However, in the absence of molecular biomarkers or neuropathological confirmation in our study, these connectivity alterations cannot be interpreted as direct evidence of tau propagation. Interestingly, recent data on cerebral perfusion and tau changes in CBS patients, after adjusting for tau cortical burden, suggest that subcortical tau pathology might also contribute to functional disconnection in connected cortical areas [41]. Longitudinal FDG and tau-PET studies in CBS patients are required to test these hypotheses.

Metabolic connectivity of subcortical regions exhibited a distinct pattern characterized by selective disconnection from cortical areas—particularly caudate and thalamic connections with sensorimotor and occipital cortices—yet relative preservation or even increases in within-subcortical connectivity, especially evident in graph-theoretical metrics. Reduced thalamic coupling with cortical regions was reported in previous fMRI- studies in CBS [22]. The caudate-cortical and basal ganglia network-level disruptions here identified are novel, suggesting greater sensitivity of metabolic connectivity analyses for detecting subcortical alterations compared to MRI-based studies. The reduced caudate-primary sensory cortex connectivity could indicate disruption of higher-order sensorimotor integration [22, 42]. Decreased correlation of caudate and putamen with the contralateral dentate indicates disruption of cerebello-basal ganglia loops. Interestingly, we observed mainly cerebellar connectivity reductions, particularly within-network disruptions, contrasting with prior MRI reports of increased cerebellar connectivity [23]. This divergence between MRI- and [^18^F]FDG-PET–based connectivity findings, with apparent great sensitivity for declines in connectivity using [^18^F]FDG-PET, likely reflect both technical and biological differences. [^18^F]FDG-PET provides a higher signal-to-noise ratio and greater statistical power, as it relies on group-level analyses [20], but it also measures a distinct biological signal [43]. Specifically, [^18^F]FDG uptake predominantly reflects astrocytic glucose metabolism supporting the glutamate–glutamine cycle at synapses [43]. In CBS, the early prominence of astrocytic plaques relative to neuronal lesions supports the hypothesis that corticobasal degeneration may begin as an astrogliopathy, with neuronal damage becoming predominant at later stages [36]. Because [^18^F]FDG uptake is influenced by astrocytic glucose metabolism supporting synaptic activity, some of the observed differences between [^18^F]FDG-PET– and MRI-based connectivity findings may relate to modality-specific biological signals. However, [^18^F]FDG-PET does not specifically measure astrocytic activity, and any interpretation regarding early astroglial dysfunction remains speculative. Longitudinal multimodal studies combining [^18^F]FDG-PET with tau and astroglial PET tracers will be needed to directly test this hypothesis.

Our graph-theoretical metrics revealed reduced cortical node strength alongside relative increases in subcortical hub strength and mixed clustering changes, indicating compensatory reorganization within subcortical networks as cortical integration declines. Findings of connectivity increases in subcortical areas are consistent with MRI evidence of enhanced dentate nucleus connectivity toward cortical regions, reported as a CBS-specific feature even compared with progressive supranuclear palsy [22]. The combination of widespread cortical connectivity reduction and relative increases in subcortical node strength likely reflects compensatory reorganization within subcortical networks. Prior studies have associated subcortical atrophy, rather than connectivity alterations, with prognosis [21], suggesting that subcortical hypometabolism may serve as a robust prognostic biomarker, whereas cortical connectivity measures offer mechanistic insights into disease propagation and compensation [21]. Moreover, the apparent discrepancy between regional and graph-theoretical metrics underscores their complementarity: regional analyses capture absolute pairwise reductions in connectivity, while graph metrics quantify the relative redistribution of nodal importance [20]. Widespread cortical disconnection can therefore yield apparent increases in subcortical nodal strength, reflecting network reorganization rather than true hyperconnectivity.

A key strength of this study is the comprehensive characterization of CBS-related brain changes using voxel-wise, ROI-based, and metabolic connectivity analyses of [^18^F]FDG-PET data, providing novel insights into cortical and subcortical network alterations. Relevant limitations include the flipping of scans to align the most affected hemisphere, which prevents full assessment of strongly lateralized regions such as language areas. Another important limitation is the absence of neuropathological confirmation or in vivo molecular biomarkers, such as amyloid PET, tau PET, or CSF biomarkers. Because CBS is a clinically defined syndrome encompassing heterogeneous underlying pathologies, the regional metabolic and connectivity alterations observed in our cohort should be interpreted in the context of the clinical syndrome rather than attributed to a specific underlying proteinopathy. Consequently, mechanistic interpretations should be made with appropriate caution. Finally, the cross-sectional design limits inferences about disease progression. Further research with larger, longitudinal, and pathologically confirmed cohorts is needed to validate and extend these findings.

## Conclusion

Together, these findings provide novel insights into the metabolic and network alterations underlying CBS, highlighting asymmetric cortical hypometabolism alongside widespread cortical connectivity decline and subcortical network reorganization. [^18^F]FDG-PET–based metabolic connectivity appears to be a sensitive tool for detecting early network dysfunction in CBS, complementing MRI-based approaches and enhancing the characterization of disease mechanisms.

## Data Availability

The datasets generated during and/or analysed during the current study are available from the corresponding author on reasonable request.
